# Compact Layers of Hybrid Halide Perovskites Fabricated via the Aerosol Deposition Process—Uncoupling Material Synthesis and Layer Formation

**DOI:** 10.3390/ma9040277

**Published:** 2016-04-08

**Authors:** Fabian Panzer, Dominik Hanft, Tanaji P. Gujar, Frank-Julian Kahle, Mukundan Thelakkat, Anna Köhler, Ralf Moos

**Affiliations:** 1 Department of Functional Materials, University of Bayreuth, Bayreuth 95440, Germany; fabian.panzer@uni-bayreuth.de (F.P.); functional.materials@uni-bayreuth.de (D.H.); 2 Experimental Physics II, University of Bayreuth, Bayreuth 95440, Germany; julian.kahle@uni-bayreuth.de (F.-J.K.); anna.koehler@uni-bayreuth.de (A.K.); 3 Bayreuth Institute of Macromolecular Research (BIMF), University of Bayreuth, Bayreuth 95440, Germany; 4 Applied Functional Polymers, Macromolecular Chemistry I, University of Bayreuth, Bayreuth 95440, Germany; tanaji.gujar@uni-bayreuth.de (T.P.G.); mukundan.thelakkat@uni-bayreuth.de (M.T.)

**Keywords:** AD, room temperature impact consolidation (RTIC), methylammonium lead trihalide, MAPI_3_, CH_3_NH_3_PbI_3_, perovskite solar cell, X-ray diffraction, optical spectroscopy, scanning electron microscopy (SEM)

## Abstract

We present the successful fabrication of CH_3_NH_3_PbI_3_ perovskite layers by the aerosol deposition method (ADM). The layers show high structural purity and compactness, thus making them suitable for application in perovskite-based optoelectronic devices. By using the aerosol deposition method we are able to decouple material synthesis from layer processing. Our results therefore allow for enhanced and easy control over the fabrication of perovskite-based devices, further paving the way for their commercialization.

## 1. Introduction

Hybrid lead-halide perovskites are attracting increasing attention among various research communities due to their remarkable optoelectronic properties, which render them suitable for use as highly efficient active semiconductors in different types of devices. Up to now, their most prominent application is in perovskite-based solar cells, where efficiencies have grown from 9% in 2012 up to a remarkable and commercially interesting 21% in 2015 [1,2]. Meanwhile, applications in the field of lighting technology have also been developed. Here, different types of perovskite-based laser devices have been reported within the past two years [3,4,5,6]. Further, by embedding the perovskite material in a host matrix, perovskite-based white light light-emitting diodes were fabricated [7]. Since perovskites also absorb in higher-energy spectral regions, a successful use as UV- or X-ray detectors was demonstrated recently [8,9,10,11,12]. Additionally, hybrid halide perovskites were found to work as memory devices [13,14] and transistors [15], thus further extending the number of possible fields of application for this material class. In general, all these different types of applications impressively demonstrate the high potential of this class of materials.

Obtaining good control over the formation of the perovskite layer is a key requirement to enable the exploitation of the perovskite’s optoelectronic properties in any of these applications. As a consequence, a variety of different methods for the formation of thin films of perovskite were presented within the last years [16,17]. Most of the methods are based on the same principle. Two different compounds, where at least one of them is a halide, are combined to result in a perovskite structure. Usually, this is done in solution and the perovskite formation occurs during the drying process which leads to the film [16]. This approach has the disadvantage that synthesis of the perovskite is interconnected with the formation of the film, so that changes in the processing of the film imply concomitant changes in the optoelectronic properties of the perovskites. Furthermore, it is difficult to obtain layers with thicknesses significantly above 1 µm, which are needed, for instance, when the perovskite is used in X-ray detectors. The development of an alternative approach, where the perovskite synthesis is decoupled from the formation of the film and where films with a wide range of thicknesses can be made easily and without altering the material’s properties, is therefore highly desired. This would allow for more control over the processing of the various kinds of perovskite-based devices, further paving the way for commercialization.

A novel processing method that may satisfy the aforementioned requirements is the so-called aerosol deposition (AD) process or method. As we showed in several previous studies [18,19,20,21,22], this method, emerging from the field of ceramics engineering, has proved to be applicable to various material systems and applications. As a result, it attracted much attention over the past decade [23]. Moreover, AD was already used for the controlled formation of TiO_2_ layers in dye-sensitized solar cells (DSSCs) [24,25]. In general, it is a method that is used to produce dense ceramic coatings fully at room temperature conditions directly from a bulk powder. The powder is transferred into an aerosol and then spray-coated onto a substrate where dense films are formed. Reported film thicknesses range from a single micron up to several hundreds of micrometers, while reaching film porosities in the single percent range without an additional sintering step [23]. This makes AD superior to other manufacturing methods, especially for materials with low decomposition/degrading temperatures, as is the case in organic-inorganic hybrid perovskites.

The dry nature of the AD process is in contrast to the already-used spray-coating methods that have been applied to hybrid perovskites so far [26,27,28,29,30]. These methods have in common that the perovskite is synthesized *in situ* after *wet* deposition of the reactants, which can render control over the reaction difficult. For example, a perovskite precursor containing methylammonium iodide and lead chloride is deposited by spray-coating and the perovskite forms after annealing [7,26], or PbI_2_ and Ch_3_NH_3_I are subsequently deposited on a substrate by aerosol-assisted chemical vapor deposition and an annealing step results in perovskite formation [28,30,31]. The dry deposition of perovskite powder that we employ, however, implies that the synthesis of the perovskite and the formation of the film are, finally, detached from each other and can be optimized independently. This is a major advancement on the way to the commercialization of this class of semiconductor.

In this proof-of-principle study, we present the successful use of AD to form CH_3_NH_3_PbI_3_ perovskite layers, which show high crystalline quality, compactness and optoelectronic activity, thus making this compound suitable for use as active elements in various perovskite-based devices.

## 2. Results and Discussion

**Figure 1:**
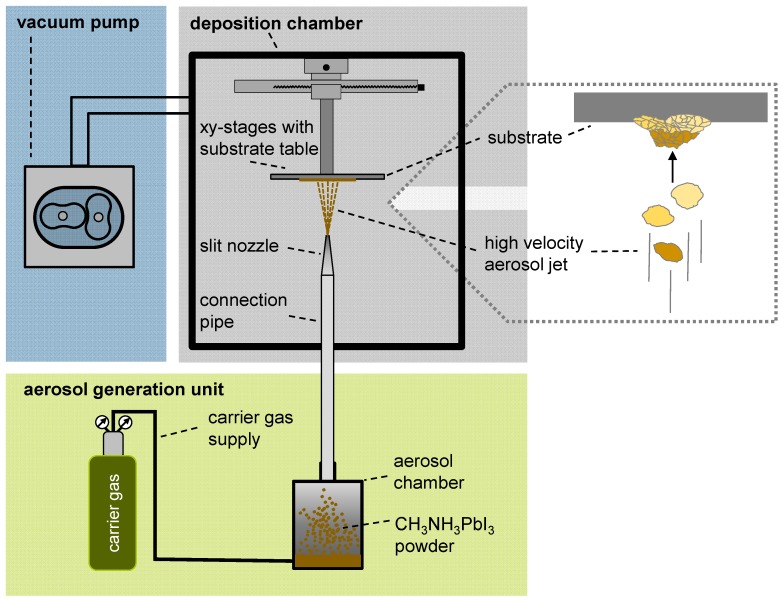
Schematic representation of an aerosol deposition setup with its typical components. The zoomed area at the right-hand side illustrates the film formation process in more detail.

A schematic illustration of the AD system that was used in this study is presented in Figure 1. It consists of three main components, a deposition chamber, a vacuum pump, and an aerosol generation unit. In the aerosol generation unit, a carrier gas flow (*i.e.*, N_2_) is directed at the perovskite powder filling which creates aerosolized particles within the aerosol chamber. Due to the pressure difference compared to the deposition chamber, which is evacuated by a vacuum pump (*ca.* 10 mbar), the perovskite particle gas flow is accelerated and dragged through a connecting pipe into the deposition chamber. A slit nozzle is mounted to the exit of the pipe for additional acceleration of the aerosol flow to form a high velocity jet. This jet is then focused toward a movable substrate, where it forms a film when the particles impact on the substrate and consolidate (Figure 1) [23,32]. Here, various parameters such as particle size, hardness of the material or velocity of the particle jet are known to affect the formation of a film processed by AD. For a detailed overview of the AD with parameters influencing film formation, materials investigated so far (*i.e.*, TiO_2_) and possible applications, we refer to the reviews [20,23]. In contrast to the related method of organic vapor-phase deposition, which has been used successfully for the fabrication of organic light-emitting diodes yet requires heating of the equipment and the carrier gas to temperatures in the range of 200–300 °C, the aerosol formation and deposition occurs at room temperature [33,34]. Thus, the principle of film formation is also called Room Temperature Impact Consolidation (RTIC) [23].

**Figure 2:**
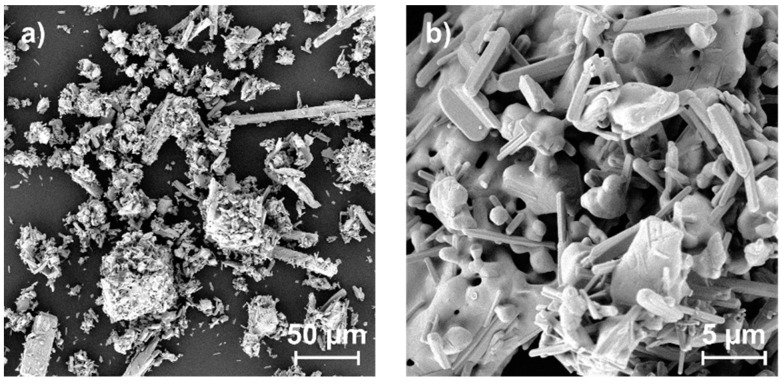
Top-view SEM images of the synthesized perovskite powder before spraying at (**a**) lower; and (**b**) higher magnification.

We prepared perovskite powder following a synthesis method as described in more detail in the Experimental Section. Here, the perovskite material was pestled to a powder as the last preparation step before spraying. Figure 2 shows an SEM top-view image of the synthesized powder. From this, a broad distribution of particle sizes in the range of submicrons up to 30 µm becomes obvious. In principle, such a wide distribution as well as the strong agglomeration of the powder particles is disadvantageous for the AD process, where a rather narrow particle size distribution in the single-micron range is usually desired [20,23]. When having a detailed view of the particles (Figure 2b), it can be seen that they consist of smaller constituents, which reveals the partially agglomerated character of the powder, which is also not advantageous for ADM. As will be explained in more detail below, it nevertheless was possible to transform the perovskite powder into a film using AD.

**Figure 3:**
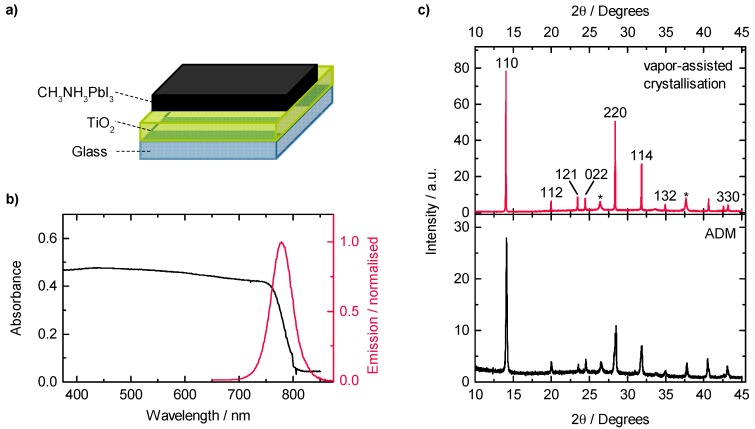
(**a**) Schematic of the sample with an AD-processed perovskite layer; (**b**) Absorption (black solid line) and normalized photoluminescence spectrum (red solid line) of the AD-processed perovskite film; (**c**) XRD patterns of a reference film produced by a vapor-assisted crystallization approach (top) and the AD-processed film (bottom).

To prove that film formation is possible on relevant interfaces, the perovskite powder was then processed in terms of the above-described AD onto a glass substrate that was covered with a TiO_2_ layer. The latter is frequently used as a transport layer in perovskite solar cells (Figure 3a) [35]. Figure 3b shows the room temperature absorption spectrum of the thus-prepared perovskite film, along with the corresponding normalized photoluminescence spectrum of the sample. The spectra exhibit the typical optical features of CH_3_NH_3_PbI_3_ [36], which is a broad absorption within the entire visible range with an absorption onset in the spectral range of about 770 nm. In emission, the samples show the typical near–band edge emission feature at about 780 nm with a FWHM of 46 nm, in accordance with reported literature values [36,37].

To further address the question on the structural quality of the processed layer, we performed XRD measurements. Figure 3c shows the XRD pattern of one of the prepared samples processed via AD, together with the XRD results of a perovskite film which was fabricated via an optimized vapor-assisted crystallization approach for comparison. The latter approach was developed recently in our group and was proven to result in highly stable, uniform and compact layers [38]. When comparing the XRD spectra, both methods lead to diffraction patterns with main features at 14.1°, 28.4°, 31.8° and 43.2° which are assigned to the 110, 220, 114 and 330 peaks of the CH_3_NH_3_PbI_3_ perovskite structure, respectively [38,39,40]. From this, a perovskite-type structure of the AD-processed layer is evident. Notably, no feature in the range of 12.6° is observed. Such a feature is commonly attributed to PbI_2_ incorporations, indicative of a non-completed perovskite formation during material synthesis or a degradation process of the perovskite [39]. Thus, the absence of such characteristic features of PbI_2_ in our spectra is formidable proof of the nondestructive character of the AD when processing lead-halide perovskite powders. In contrast to the X-ray diffraction spectrum of the layer produced by vapor-assisted crystallization, the X-ray diffraction features of the AD-processed sample generally show less intensity and, simultaneously, a broader width of the peaks. From both of these observations a smaller average grain size can be concluded [41].

**Figure 4:**
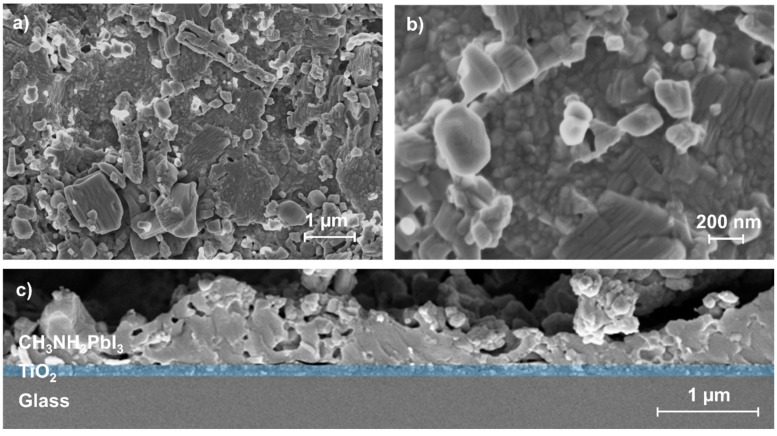
(**a**) Top-view SEM images of the AD-processed perovskite layer with a lower; and (**b**) higher magnification; and (**c**) fracture cross-section of the AD-processed substrate with the TiO_2_ layer highlighted in blue.

This becomes further evident when considering top-view SEM images (Figure 4). Here we find a wide distribution of grain sizes ranging from below 100 nm up to 1000 nm in the AD-processed film (Figure 4a). This is smaller than the reported grain sizes for the optimized vapor-assisted crystallization method, which are in the range between 500 nm and 2000 nm. We assume the rather wide distribution of grain sizes in the case of the AD-processed layers to be due to the distribution of particle sizes of the perovskite powder on the one hand, as no classifying or filtering treatments were applied to the source material. On the other hand, the crystallite size in the 20 to 50 nm range, as can be seen in Figure 4b, can be attributed to the RTIC film formation mechanism that typically governs the AD process. Here, the high kinetic energy of impacting particles results in their fracturing and in a consolidation of previously deposited particles by hammering.

Figure 4b shows that the processed layer exhibits a compact character within the investigated area which is an important layer property for highly efficient optoelectronic applications. For the latter, another important requirement is an intimate contact between the perovskite and TiO_2_. From a fracture cross-sectional SEM image of the AD-processed layer (Figure 4c), such a direct contact between the two components can also be seen in our case. Therefore, from the data in Figure 3 and Figure 4 we conclude that AD can transfer the source material to a layer without destroying the crystal structure. From Figure 4c, the layer structure can be characterized as primarily dense with an occasionally distributed number of flaws. We attribute the latter to result from the completely untreated and thus not-for-AD-optimized nature of the initial powder (see Figure 2). Thus, an optimization of the initial powder is an apparent starting point to improve the overall process. This may be achieved by powder preparation methods as they are typical for ceramics, *i.e.*, ball-milling or attritor milling.

In summary, we have shown the successful and nondestructive deposition of lead-halide perovskite CH_3_NH_3_PbI_3_ powders leading to a compact layer with high chemical purity. The aerosol deposition method employed decouples the synthesis of the perovskite from the layer formation process. It is well established that aerosol deposition allows for the fabrication of a wide range of layer thicknesses [20], that it can also be employed to deposit Al_2_O_3_ [18] and TiO_2_ [25], which are frequently used as transport layers in perovskite solar cells, and that it lends itself to the deposition of mixtures of different source materials [22,42,43,44]. Thus, this method has potential for a range of different perovskite-based solar cell device architectures. Moreover, this method is scalable and thus is suited for rapid, high-throughput deposition and patterning, as required in an industrial context.

## 3. Materials and Methods

Materials: All materials were purchased from Sigma-Aldrich and used as received.

CH_3_NH_3_I Synthesis: Methylammonium iodide (MAI) was synthesized as discussed elsewhere [38]. In short, MAI was synthesized by reacting 24 mL of methylamine (33 wt. % in absolute ethanol) and 10 mL of hydroiodic acid (57 wt. % in water) in a round-bottom flask at 0 °C for 2 h with stirring. The precipitate was recovered by putting the solution on a rotary evaporator and carefully removing the solvents at 50 °C. The white raw product MAI was re-dissolved in 80 mL absolute ethanol and precipitate with the addition diethyl ether. After filtration, the step was repeated two times and white solid was collected and dried at 60 °C in a vacuum oven for 24 h.

Preparation of CH_3_NH_3_PbI_3_ powder: The MAI and Lead(II) iodide (PbI_2_) were mixed together with 1:1 ratio in 2 mL N, N-dimethyl formamide (DMF) in round-bottom flask. The mixture was stirred for 30 min and degassed under N_2_ gas for 30 min followed by drying under N_2_ atmosphere at 100 °C. Finally, the dried powder was collected and ground using mortar.

Film Deposition: For deposition we used a custom-made AD apparatus. We used a 10 mm slit nozzle to prepare films of 10 × 15 mm² area on the TiO_2_-coated glass substrate.

Layer characterization: Absorption spectrum at room temperature was measured in an integrating sphere using a Cary 5000 UV/Vis spectrometer from Varian (Santa Clara, CA, USA). For emission measurement, we used a FP-8600 spectral photometer from JASCO where the sample was excited at a wavelength of 405 nm. The film was examined by X-ray diffraction (XRD), using a Bruker D8 Advance, with Cu Kα (*λ* = 1.5406) X-Ray source. The scanning was conducted in the range of 2*θ* = 10°–45°, with a step size of 0.008° and at a rotation speed of 15 min^−1^. The generator voltage and current were set to 40 kV and 40 mA, respectively. The surface morphology was characterized by field emission scanning electron microscopy (FE-SEM) using a Zeiss 1530 instrument (Zeiss, Oberkochen, Germany).with an accelerating voltage of 3.0 kV.

## References

[1] NREL Solar Efficiency Chart. http://www.nrel.gov/ncpv/images/efficiency_chart.jpg.

[2] Luo S., Daoud W. (2016). Crystal Structure Formation of CH_3_NH_3_PbI_3-x_Cl_x_ Perovskite. Materials.

[3] Deschler F., Price M., Pathak S., Klintberg L.E., Jarausch D.D., Higler R., Hüttner S., Leijtens T., Stranks S.D., Snaith H.J. (2014). High photoluminescence efficiency and optically pumped lasing in solution-processed mixed halide perovskite semiconductors. J. Phys. Chem. Lett..

[4] Zhu H., Fu Y., Meng F., Wu X., Gong Z., Ding Q., Gustafsson M.V., Trinh M.T., Jin S., Zhu X.Y. (2015). Lead halide perovskite nanowire lasers with low lasing thresholds and high quality factors. Nat. Mater..

[5] Stranks S.D., Wood S.M., Wojciechowski K., Deschler F., Saliba M., Khandelwal H., Patel J.B., Elston S.J., Herz L.M., Johnston M.B. (2015). Enhanced amplified spontaneous emission in perovskites using a flexible cholesteric liquid crystal reflector. Nano Lett..

[6] Saliba M., Wood S.M., Patel J.B., Nayak P.K., Huang J., Alexander-Webber J.A., Wenger B., Stranks S.D., Horantner M.T., Wang J.T. (2016). Structured organic-inorganic perovskite toward a distributed feedback laser. Adv. Mater..

[7] Pathak S., Sakai N., Wisnivesky Rocca Rivarola F., Stranks S.D., Liu J., Eperon G.E., Ducati C., Wojciechowski K., Griffiths J.T., Haghighirad A.A. (2015). Perovskite Crystals for tunable white light emission. Chem. Mater..

[8] Náfrádi B., Náfrádi G., Forró L., Horváth E. (2015). Methylammonium lead iodide for efficient X-ray energy conversion. J. Phys. Chem. C.

[9] Maculan G., Sheikh A.D., Abdelhady A.L., Saidaminov M.I., Haque M.A., Murali B., Alarousu E., Mohammed O.F., Wu T., Bakr O.M. (2015). CH_3_NH_3_PbCl_3_ Single Crystals: Inverse Temperature Crystallization and Visible-Blind UV-Photodetector. J. Phys. Chem. Lett..

[10] Yakunin S., Sytnyk M., Kriegner D., Shrestha S., Richter M., Matt G.J., Azimi H., Brabec C.J., Stangl J., Kovalenko M.V. (2015). Detection of X-ray photons by solution-processed lead halide perovskites. Nat. Photonics.

[11] Wang F., Mei J., Wang Y., Zhang L., Zhao H., Zhao D. (2016). Fast photoconductive responses in organometal halide perovskite photodetectors. ACS Appl. Mater. Interfaces.

[12] Ramasamy P., Lim D.H., Kim B., Lee S.H., Lee M.S., Lee J.S. (2016). All-inorganic cesium lead halide perovskite nanocrystals for photodetector applications. Chem. Commun..

[13] Yoo E.J., Lyu M., Yun J.H., Kang C.J., Choi Y.J., Wang L. (2015). Resistive switching behavior in organic-inorganic hybrid CH_3_NH_3_PbI_3-x_Cl_x_ perovskite for resistive random access memory devices. Adv. Mater..

[14] Panzer F., Baderschneider S., Gujar T.P., Unger T., Bagnich S., Jakoby M., Bässler H., Hüttner S., Köhler J., Moos R. (2016). Reversible laser induced amplified spontaneous emission from coexisting tetragonal and orthorhombic phases in hybrid lead halide perovskites. Adv. Opt. Mater..

[15] Chin X.Y., Cortecchia D., Yin J., Bruno A., Soci C. (2015). Lead iodide perovskite light-emitting field-effect transistor. Nat. Commun..

[16] Stranks S.D., Nayak P.K., Zhang W., Stergiopoulos T., Snaith H.J. (2015). Formation of thin films of organic-inorganic perovskites for high-efficiency solar cells. Angew. Chem. Int. Ed..

[17] Ono L.K., Leyden M.R., Wang S., Qi Y. (2016). Organometal halide perovskite thin films and solar cells by vapor deposition. J. Mater. Chem. A.

[18] Schubert M., Exner J., Moos R. (2014). Influence of carrier gas composition on the stress of Al_2_O_3_ coatings prepared by the aerosol deposition method. Materials.

[19] Sahner K., Kaspar M., Moos R. (2009). Assessment of the novel aerosol deposition method for room temperature preparation of metal oxide gas sensor films. Sens. Actuators B Chem..

[20] Hanft D., Exner J., Schubert M., Stöcker T., Fuierer P., Moos R. (2015). An overview of the aerosol deposition method: Process fundamentals and new trends in materials applications. J. Ceram. Sci. Technol..

[21] Exner J., Hahn M., Schubert M., Hanft D., Fuierer P., Moos R. (2015). Powder requirements for aerosol deposition of alumina films. Adv. Powder Technol..

[22] Exner J., Fuierer P., Moos R. (2014). Aerosol deposition of (Cu,Ti) substituted bismuth vanadate films. Thin Solid Films.

[23] Akedo J. (2008). Room temperature impact consolidation (RTIC) of fine ceramic powder by aerosol deposition method and applications to microdevices. J. Therm. Spray Technol..

[24] Yang S., Kim H., Ahn S.-H., Lee C.S. (2015). The effect of the agglomerated microstructure of dry-deposited TiO_2_ electrodes on the performance of dye-sensitized solar cells. Electrochim. Acta.

[25] Cho S.H., Yoon Y.J. (2013). Multi-layer TiO_2_ films prepared by aerosol deposition method for dye-sensitized solar cells. Thin Solid Films.

[26] Bhachu D.S., Scanlon D.O., Saban E.J., Bronstein H., Parkin I.P., Carmalt C.J., Palgrave R.G. (2015). Scalable route to CH_3_NH_3_PbI_3_ perovskite thin films by aerosol assisted chemical vapour deposition. J. Mater. Chem. A.

[27] Barrows A.T., Pearson A.J., Kwak C.K., Dunbar A.D.F., Buckley A.R., Lidzey D.G. (2014). Efficient planar heterojunction mixed-halide perovskite solar cells deposited via spray-deposition. Energy Environ. Sci..

[28] Chen S., Briscoe J., Shi Y., Chen K., Wilson R.M., Dunn S., Binions R. (2015). A simple, low-cost CVD route to high-quality CH_3_NH_3_PbI_3_ perovskite thin films. Cryst. Eng. Comm..

[29] Ishihara H., Sarang S., Chen Y.C., Lin O., Phummirat P., Thung L., Hernandez J., Ghosh S., Tung V. (2016). Nature inspiring processing route toward high throughput production of perovskite photovoltaics. J. Mater. Chem. A.

[30] Lewis D.J., O’Brien P. (2014). Ambient pressure aerosol-assisted chemical vapour deposition of (CH_3_NH_3_)PbBr_3_, an inorganic-organic perovskite important in photovoltaics. Chem. Commun..

[31] Leyden M.R., Ono L.K., Raga S.R., Kato Y., Wang S., Qi Y. (2014). High performance perovskite solar cells by hybrid chemical vapor deposition. J. Mater. Chem. A.

[32] Lee D.W., Kim H.J., Kim Y.H., Yun Y.H., Nam S.M. (2011). Growth process of α-Al_2_O_3_ ceramic films on metal substrates fabricated at room temperature by aerosol deposition. J. Am. Ceram. Soc..

[33] Arnold M.S., McGraw G.J., Forrest S.R., Lunt R.R. (2008). Direct vapor jet printing of three color segment organic light emitting devices for white light illumination. Appl. Phys. Lett..

[34] McGraw G.J., Forrest S.R. (2013). Vapor-Phase Microprinting of Multicolor Phosphorescent Organic Light Emitting Device Arrays. Adv. Mater..

[35] Shi S.W., Li Y.F., Li X.Y., Wang H.Q. (2015). Advancements in all-solid-state hybrid solar cells based on organometal halide perovskites. Mater. Horiz..

[36] Galisteo-Lopez J.F., Anaya M., Calvo M.E., Miguez H. (2015). Environmental effects on the photophysics of organic-inorganic halide perovskites. J. Phys. Chem. Lett..

[37] Stranks S.D., Eperon G.E., Grancini G., Menelaou C., Alcocer M.J., Leijtens T., Herz L.M., Petrozza A., Snaith H.J. (2013). Electron-hole diffusion lengths exceeding 1 micrometer in an organometal trihalide perovskite absorber. Science.

[38] Gujar T.P., Thelakkat M. (2016). Highly reproducible and efficient perovskite solar cells with extraordinary stability from robust CH_3_NH_3_PbI_3_: Towards large-area devices. Energy Technol..

[39] Liu M., Johnston M.B., Snaith H.J. (2013). Efficient planar heterojunction perovskite solar cells by vapour deposition. Nature.

[40] Chen Q., Zhou H., Hong Z., Luo S., Duan H.S., Wang H.H., Liu Y., Li G., Yang Y. (2014). Planar heterojunction perovskite solar cells via vapor-assisted solution process. J. Am. Chem. Soc..

[41] Bellet D., Bellet-Amalric E. (2014). Physical Characterisation of Photovoltaic Materials. Solar Cell Materials.

[42] Exner J., Fuierer P., Moos R. (2014). Aerosol codeposition of ceramics: Mixtures of Bi_2_O_3_-TiO_2_ and Bi_2_O_3_-V_2_O_5_. J. Am. Ceram. Soc..

[43] Park J.H., Akedo J., Nakada M. (2006). Surface plasmon resonance in novel nanocomposite gold/lead zirconate titanate films prepared by aerosol deposition method. Jpn. J. Appl. Phys..

[44] Ryu J., Hahn B.D., Choi J.J., Yoon W.H., Lee B.K., Choi J.H., Park D.S. (2010). Porous photocatalytic TiO_2_ thin films by aerosol deposition. J. Am. Ceram. Soc..

